# Multidimensional Feature Tuning in Category Selective Areas of Human Visual Cortex

**DOI:** 10.1523/JNEUROSCI.0038-26.2026

**Published:** 2026-06-17

**Authors:** Leonard E. van Dyck, Martin N. Hebart, Katharina Dobs

**Affiliations:** ^1^Department of Computer Science, Justus Liebig University Giessen, Giessen 35390, Germany; ^2^Max Planck Institute for Human Cognitive and Brain Sciences, Leipzig 04103, Germany; ^3^Department of Medicine, Justus Liebig University Giessen, Giessen 35390, Germany; ^4^Center for Mind, Brain and Behavior (CMBB), Universities of Marburg, Giessen, and Darmstadt, Marburg 35032, Germany

**Keywords:** category selectivity, dimensions, distributed processing, fMRI, functional organization, visual cortex

## Abstract

Two prominent accounts describe the functional organization of human high-level visual cortex. A categorical view emphasizes category-selective areas, while a dimensional view highlights continuous feature maps spanning these areas. Here, we asked whether these two views reflect complementary expressions of the same underlying organization. Using a data-driven decomposition of fMRI responses from human participants (female and male) in face-, body-, and scene-selective areas, we identified spatially overlapping activity patterns that were shared across individuals. Each area encoded multiple interpretable dimensions capturing both finer within-category and coarser between-category distinctions, even in the most category-selective voxels. These dimensions formed distinct clusters within category-selective areas but extended as distributed maps across visual cortex. Together, these findings reveal an underlying organization that links category-selective areas to continuous feature maps, thereby reconciling categorical and dimensional accounts of high-level visual cortex.

## Significance Statement

What functional organization in human visual cortex could give rise to both category-selective areas and continuous feature maps? Using a data-driven decomposition of fMRI responses to natural images, we identified interpretable dimensions that explain activity in face-, body-, and scene-selective areas. These dimensions captured both within-category and between-category distinctions, formed distinct clusters within category-selective areas, and extended as distributed maps across visual cortex. This underlying functional organization reconciles categorical and dimensional accounts and helps explain how visual cortex balances specificity and flexibility to support diverse perceptual and cognitive demands.

## Introduction

Human visual cortex contains category-selective areas that respond preferentially to specific visual inputs, such as faces, bodies, and scenes (Kanwisher et al., 1997; Epstein and Kanwisher, 1998; Downing et al., 2001; Kanwisher, 2010). The discovery of these specialized areas gave rise to a categorical view of cortical organization, in which category membership serves as the primary organizing principle of high-level visual cortex, encompassing ventral and lateral occipito-temporal cortex beyond early retinotopic maps. This view is supported by univariate analyses that contrast fMRI responses to predefined stimulus categories and have been instrumental in identifying classical category-selective areas (Kanwisher and Yovel, 2006; Peelen and Downing, 2007; Epstein and Baker, 2019), uncovering novel category-selective responses (McCandliss et al., 2003; Bracci et al., 2010; Abassi and Papeo, 2024; Cortinovis et al., 2025; Henderson et al., 2025), and establishing their behavioral relevance (Moro et al., 2008; Kanwisher and Barton, 2011; Cohen et al., 2017).

In contrast, a dimensional view proposes that high-level visual cortex is organized along continuous feature dimensions that span categorical boundaries (Haxby et al., 2001, 2011; Bao et al., 2020; Bracci and Op de Beeck, 2023). This view has been supported by both univariate and multivariate analyses, which show that category-selective areas encode a variety of features beyond their preferred categories (Haxby et al., 2001; Cichy et al., 2012). Within these areas, functional clusters represent both finer within-category distinctions (Orlov et al., 2010; Bracci et al., 2015; de Haas et al., 2021) and coarser between-category distinctions (Arcaro et al., 2009; Çukur et al., 2013, 2016; Çelik et al., 2021), indicating a more complex and spatially overlapping organization. More broadly, neural responses in visual cortex reflect a wide range of feature dimensions, including animacy, real-world size, aspect ratio, behavioral aspects, and semantic content (Huth et al., 2012; Konkle and Oliva, 2012; Konkle and Caramazza, 2013; Watson et al., 2017; Bao et al., 2020; Almeida et al., 2023; Coggan and Tong, 2023; Abdel-Ghaffar et al., 2024; Arcaro and Livingstone, 2024; Contier et al., 2024; Watson and Andrews, 2024). These features are encoded in large-scale maps that embed category-selective areas within a continuous representational landscape.

Whether these two views are truly opposing or reflect different expressions of a common underlying organization remains debated (Op de Beeck et al., 2008; Mahon and Caramazza, 2009, 2011; Grill-Spector and Weiner, 2014; Peelen and Downing, 2017; Yargholi and de Beeck, 2023; Ritchie et al., 2026). In particular, it remains unclear what features are encoded within category-selective areas, how those features are spatially arranged across cortex, and what organizing principle could account for both category-selective areas and continuous feature maps.

To address this gap, we applied a data-driven decomposition of fMRI responses (Khosla et al., 2022) in face-, body-, and scene-selective areas during natural image viewing (Allen et al., 2022), aiming to uncover their underlying representational dimensions. Our approach builds on two theoretical assumptions (Hebart et al., 2020): (1) neural representations are sparse, such that a limited number of dimensions can capture the response to a given stimulus, and (2) these dimensions are continuous and non-negative, supporting interpretability by ensuring that they contribute additively to the overall response. To implement these assumptions, we employed non-negative matrix factorization (NMF), a method that identifies part-based, sparse, and interpretable components. This approach allowed us to directly test the two views against each other and ask what underlying organization could give rise to both.

By analyzing both the representational content and cortical topography of the identified dimensions, we found evidence supporting essential aspects of both accounts. Category-selective areas encoded information using dimensions that were consistent across individuals. Most dimensions encoded features related to their preferred category, but some also encoded nonpreferred features, revealing multifaceted representations even in the most category-selective voxels. The dimensions formed distinct clusters within category-selective areas and extended as distributed maps across cortex. Together, these findings reveal an underlying organization that links category-selective areas to continuous feature maps, thereby reconciling categorical and dimensional views in high-level visual cortex.

## Materials and Methods

### fMRI data

#### Natural Scenes Dataset

We used the Natural Scenes Dataset (NSD; Allen et al., 2022), which includes fMRI responses from eight participants (female and male) who each viewed 9,000–10,000 natural scene images over 30–40 scan sessions, with each image repeated three times. Our analyses focused on participants who completed all trials (P1: S1, P2: S2, P3: S5, and P4: S7), viewing 9,000 unique and 1,000 shared images. During scanning, participants maintained central fixation and performed a long-term recognition task by identifying previously viewed images. Images were sourced from Microsoft Common Objects in Context (COCO; Lin et al., 2014) and displayed at a visual angle of 8.4° × 8.4° for 3 s, with a 1 s interval between images. BOLD responses were acquired at 7 T using whole-brain gradient-echo EPI with 1.8 mm isotropic voxel resolution and a 1.6 s repetition time. Preprocessing included temporal and spatial interpolation for slice timing and head motion correction, and single-trial voxel responses were estimated using a general linear model. We used preprocessed single-trial voxel responses optimized through voxel-wise hemodynamic response function modeling, data-driven denoising, and ridge regression (“betas_fithrf_GLMdenoise_RR”; 1.8 mm isotropic native preparation; Prince et al., 2022). Cortical flat maps were generated using PyCortex (Gao et al., 2015). Details on scanning protocols, preprocessing steps, and noise ceiling estimation are provided in the original NSD paper (Allen et al., 2022). To cross-validate our analyses, we randomly divided the images into a 70% training set and a 30% test set, ensuring that all shared images were assigned to the training set. To account for session effects and improve reliability, single-trial voxel responses were *z*-scored within each session using the mean and standard deviation of the training trials, and neural response estimates were averaged across repetitions of each image.

#### Category-selective areas

We extracted voxel responses from three functional regions of interest (ROIs): fusiform face area (FFA), parahippocampal place area (PPA), and extrastriate body area (EBA). These ROIs were identified using a separate functional localizer (fLoc; Stigliani et al., 2015). During the fLoc, participants viewed grayscale images of five categories (faces, bodies, places, characters, objects) presented in a miniblock design across six runs, with each block containing eight images per category. After standard preprocessing, category-specific beta values were estimated using a general linear model. ROIs were defined by conducting *t* tests comparing each category to all others (*t* > 2), isolating voxels with clear category preferences. Details on the analyses are provided in the original fLoc paper (Stigliani et al., 2015).

To ensure reliable input for Bayesian non-negative matrix factorization (BNMF), we selected voxels within each ROI that had a signal-to-noise ratio >0.2. We also performed the BNMF procedure for additional face-, body-, and scene-selective ROIs, including occipital face area (OFA), fusiform body area (FBA), and occipitotemporal place area (OPA). These analyses yielded results similar to those observed in the primary ROIs (FFA, EBA, and PPA). For reasons of brevity, we focus on the three primary ROIs here.

#### Voxel-wise category selectivity index

To quantify the category selectivity of each voxel, we used a *d′* index (Eq. 1) based on each ROI’s preferred category contrast (i.e., preferred category vs all other categories), where *µ*_v,c_ and *µ*_v,o_ are the mean responses of voxel *v* to category *c* and to all other categories *o* and *σ*^2^_v,c_ and *σ*^2^_v,o_ are the corresponding response variances:

$$d_{v,c}^{\prime }=\frac{\mu_{v,c}-\mu_{v,o}}{\sqrt{(\sigma_{v,c}^{2}+\sigma_{v,o}^{2})/2}}.\;(1)$$

### Data-driven voxel decomposition

#### Bayesian non-negative matrix factorization

We applied BNMF to decompose voxel responses from ROIs into dimensions, following prior work by Khosla et al. (2022). NMF decomposes a data matrix *X* (*I* × *V*), where *I* is the number of images and *V* is the number of voxels, into the product of two lower-rank matrices: a response matrix *W* (*I* × *D*) and a weight matrix *H* (*D* × *V*), where *D* is the number of dimensions, such that the product of these matrices optimally reconstructs the data. In this formulation, each column of *W* (*w*_d_) represents a dimension's response profile across images, while each row of *H* (*h*_d_) encodes its spatial map across voxels. The non-negativity constraint in NMF restricts dimensions to additive combinations, promoting part-based, sparse, and interpretable dimensions. Compared with other dimensionality reduction techniques like principal component analysis or cluster analysis, NMF offers several advantages: (1) voxel responses are represented exclusively by additive dimensions, avoiding cancellation effects; (2) voxels are not assigned only to individual dimensions or individual clusters, allowing for mixed selectivity; and (3) dimensions are sparse, with each voxel associated with only a subset of dimensions.

We applied BNMF, an extension of NMF, which incorporates Bayesian inference (Eq. 2) by imposing exponential priors on *W* (Eq. 3) and *H* (Eq. 4), and a normal likelihood for the residual matrix *E* (*I* × *V*) with noise variance *σ^2^*. BNMF employs Gibbs sampling to iteratively sample from the posterior distributions of *W*, *H*, and *σ^2^*. This Bayesian formulation yields probabilistic estimates that explicitly model noise in the data, improving robustness:

$$p(W,H,E)∼\;\prod_{i=1}^{I}\;\prod_{v=1}^{V}\;N(X_{i,v};{\left(WH\right)}_{i,v},\sigma^{2}),\;(2)$$

$$p(W)∼\;\prod_{i=1}^{I}\;\prod_{d=1}^{D}\;\mathrm{Exp}(-\rho_{i,d}W_{i,d}),\;\;W_{i,d}>0,\;(3)$$

$$p(H)∼\;\prod_{d=1}^{D}\;\prod_{v=1}^{V}\;\mathrm{Exp}(-\gamma_{d,v}H_{d,v}),\;\;H_{d,v}>0.\;(4)$$

We ran 3,000 Gibbs iterations, discarded the first 1,000 as burn-in, and retained every fifth draw afterward. The retained samples were averaged element-wise to obtain the posterior mean estimates of the factor matrices. Each model was initialized with a unique random seed, and convergence was achieved once the reconstruction error fell below 1 × 10^−5^. Details of the algorithm are provided in the original BNMF paper (Schmidt et al., 2009). To enforce non-negativity, we baseline-shifted *X* by subtracting the minimum *z*-scored response across all voxels in each ROI. To prevent data leakage, both training and test sets were shifted using the global training minimum. Any negative outliers remaining in the test set were set to 0. The constant offset introduced between training and test distributions was accounted for in subsequent encoding models by fitting an intercept term.

#### Estimating optimal dimensionality

We determined the optimal dimensionality k* for each participant’s ROI using bi-cross-validation, which accounts for the interdependence of rows and columns in matrix factorization (Owen and Perry, 2009). The data matrix *X* was shuffled and divided into four blocks: *A*, *B*, *C*, and *D*. BNMF was trained on the training block *D*, and pseudo-inverse matrices of *W* and *H* from blocks *B* and *C* were used to predict the held-out test block *A*. This process was repeated five times with random block partitions in each iteration for ranks between 1 and 100 in steps of 3, and the average prediction error across iterations was used to calculate the final cross-validation error. For each participant, k* was selected as the rank that minimized the test error. Estimating the optimal dimensionality for fMRI responses to natural images is challenging due to their hierarchical nature, which requires capturing meaningful dimensions while avoiding overfitting to spurious ones. Our approach balanced these considerations, ensuring optimal latent dimensionality within each participant’s ROI, maximizing representational capacity, while also minimizing the risk of overfitting.

#### Consensus approach

We identified dimensions within each ROI using a two-step approach to ensure reliability and generalizability. First, to address the sensitivity of BNMF to initialization, we adapted a consensus approach outlined in previous work (Kotliar et al., 2019). For each ROI, we performed 100 randomly initialized BNMF voxel decompositions with k* dimensions. Each dimension was normalized to an *L*_2_-norm of 1. Unreliable runs were removed based on a density-based outlier detection approach. To aggregate reliable dimensions across runs, we applied *k*-medoids clustering with cosine distance, using k* clusters. Cluster medians were then used as consensus dimensions for subsequent analyses. Second, to identify consistent dimensions across participants, we used a pairwise correlation approach also outlined in previous work (Khosla et al., 2022). We computed pairwise correlations between all combinations of dimensions across participants (k*_1_ × k*_2_ × k*_3_ × k*_4_). Using a greedy selection approach, we iteratively matched dimensions with the highest pairwise correlations until no more dimensions could be matched across all participants. Dimensions with a mean pairwise correlation of *r* > 0.3 were considered consistent, resulting in 8–20 consensus dimensions per ROI. This two-step approach ensured that the identified dimensions were reliable across runs and consistent across participants, providing a robust framework for subsequent analyses. To evaluate the generalizability of the dimensions, we projected the test set into the learned embedding using non-negative least squares regression.

### Data-driven dimension labeling

#### Behavioral labeling experiment

To interpret the dimensions, we conducted an online behavioral experiment. Participants (*N* = 38) were recruited via the university’s internal experiment platform. For each dimension, participants viewed a collage showing the 20 highest-scoring images combined across fMRI participants (Fig. S1). For each collage, participants were asked to provide 3–5 candidate labels that best described what the images had in common. Participants were instructed to provide labels that (1) refer to visible elements that appeared in most or all images (e.g., objects, scenes, behaviors, attributes), (2) consist of 1–3 concrete words, and (3) complete the sentence “The images show…” to ensure compatibility with the subsequent data-driven labeling procedure. We translated the German labels into English using the Google Translate API. Then, we manually removed nonsensical or overly generic labels (e.g., “colorful images”) and converted singular labels to plural (e.g., “face” to “faces”).

#### Deep learning-based label selection

To derive representative labels for each dimension, we used CLIP-ViT, a multimodal transformer model trained on image-text pairs (Radford et al., 2021). We first pooled all participant-generated labels across dimensions to form a comprehensive candidate label set. Candidate labels were embedded with CLIP-ViT-L/14 using 15 different generic prompt templates (e.g., “a picture of …”, “an image of …”, “a photo of …”) to improve semantic robustness, and image embeddings were computed for all training images. For each dimension, we quantified how well each label captured its response profile by computing the cosine similarity between the label embedding and the embeddings of all images, weighting each image by the dimension's response profile. For each label, we then averaged these similarities across prompts and subtracted the label's global mean similarity across all dimensions to reduce global semantic biases. The resulting values were then *z*-scored across labels within each dimension, emphasizing labels that were selectively associated with that dimension and down-weighting labels with consistently high similarity across dimensions. Finally, we used these *z*-scored similarities to rank labels for each dimension and visualized the highest-scoring labels as word clouds. This procedure yielded concise, semantically meaningful labels for each dimension that reflected recurring patterns in the highest-scoring images and were validated across the full image set.

#### Voxel-wise encoding models

To evaluate prediction performance and derive functional tuning maps for each ROI's dimensions, we fit a voxel-wise encoding model using the dimension response profiles to model voxel responses across cortex. Notably, predictors were based on the consistent BNMF dimensions learned from baseline-shifted ROI voxel responses, whereas the encoding model was trained to predict unshifted voxel responses to new images.

To avoid overfitting and mitigate multicollinearity among dimensions, we used fractional ridge regression (Rokem and Kay, 2020). This regularization technique interpolates between the ordinary least squares solution (fraction = 1, no regularization) and maximal regularization (fraction = 0, coefficients shrunk to zero). For each voxel, the ridge fraction was optimized with 10-fold cross-validation on the training set. Within each fold, fractions were tuned on 90% of the training data and validated on the remaining 10%, sampled from 0.1 to 0.9 in increments of 0.1, and from 0.9 to 1 in increments of 0.01 for higher precision in the less regularized range. Predictors were *z*-scored within folds, and an intercept was included to account for the baseline shift. We calculated prediction performance (*R*^2^) for each fold and selected the fraction that yielded the highest average performance per voxel. Using these voxel-wise optimal fractions, we refit the model on the full training set and evaluated it on the held-out test set by correlating predicted and observed voxel responses.

To obtain dimension response profiles for the test set, we projected ROI voxel responses to the test images onto the BNMF spatial maps learned from the training data. Holding the spatial maps fixed, we used non-negative least squares to estimate non-negative weights for each test image.

Statistical significance was assessed by generating a null distribution of correlations using 3,000 random permutations of the test set within each fold. Voxel-wise *p* values were calculated using a one-tailed *t* test and corrected for multiple comparisons using the Benjamini–Hochberg procedure with a false discovery rate (FDR) of *p* < 0.01. This framework preserves non-negative dimension response profiles while allowing voxel coefficients to be positive or negative, enabling identification of cortical areas whose tuning is negatively related to a given dimension. A strong linear relationship between the spatial mappings from BNMF and the encoding model confirmed that relaxing the non-negativity constraint did not compromise the validity of the original weights (mean *r* across participants ± SD; FFA: *r* = 0.94 ± 0.04; EBA: *r* = 0.89 ± 0.05; PPA: *r* = 0.89 ± 0.06). Noise ceilings were computed for the test set following the methodology provided in the original NSD paper (Allen et al., 2022).

#### Representational sparseness

We quantified the extent of multidimensional tuning using a measure of representational sparseness based on the normalized relationship between the *L*_1_- and *L*_2_-norm of a vector (Hoyer, 2004; Eq. 5). For each voxel, the dimension weights of the encoding model were interpreted as its *n*-dimensional tuning profile *x*. The resulting sparseness index *s* ranges from 0, indicating a perfectly dense representation tuned equally to all dimensions, to 1, indicating a perfectly sparse representation tuned to a single dimension. We used this measure to quantify relative differences in tuning concentration across voxels within a given area, but refrain from comparing absolute sparseness levels across areas, as the non-negativity constraint imposed by NMF may favor sparser solutions overall.

$$s(x)=\frac{\sqrt{n}-\sum |x_{i}|/\sqrt{\sum x_{i}^{2}}}{\sqrt{n}-1}.\;(5)$$

## Results

### Data-driven voxel decomposition reveals multiple interpretable dimensions in category-selective areas

**Figure 1.**
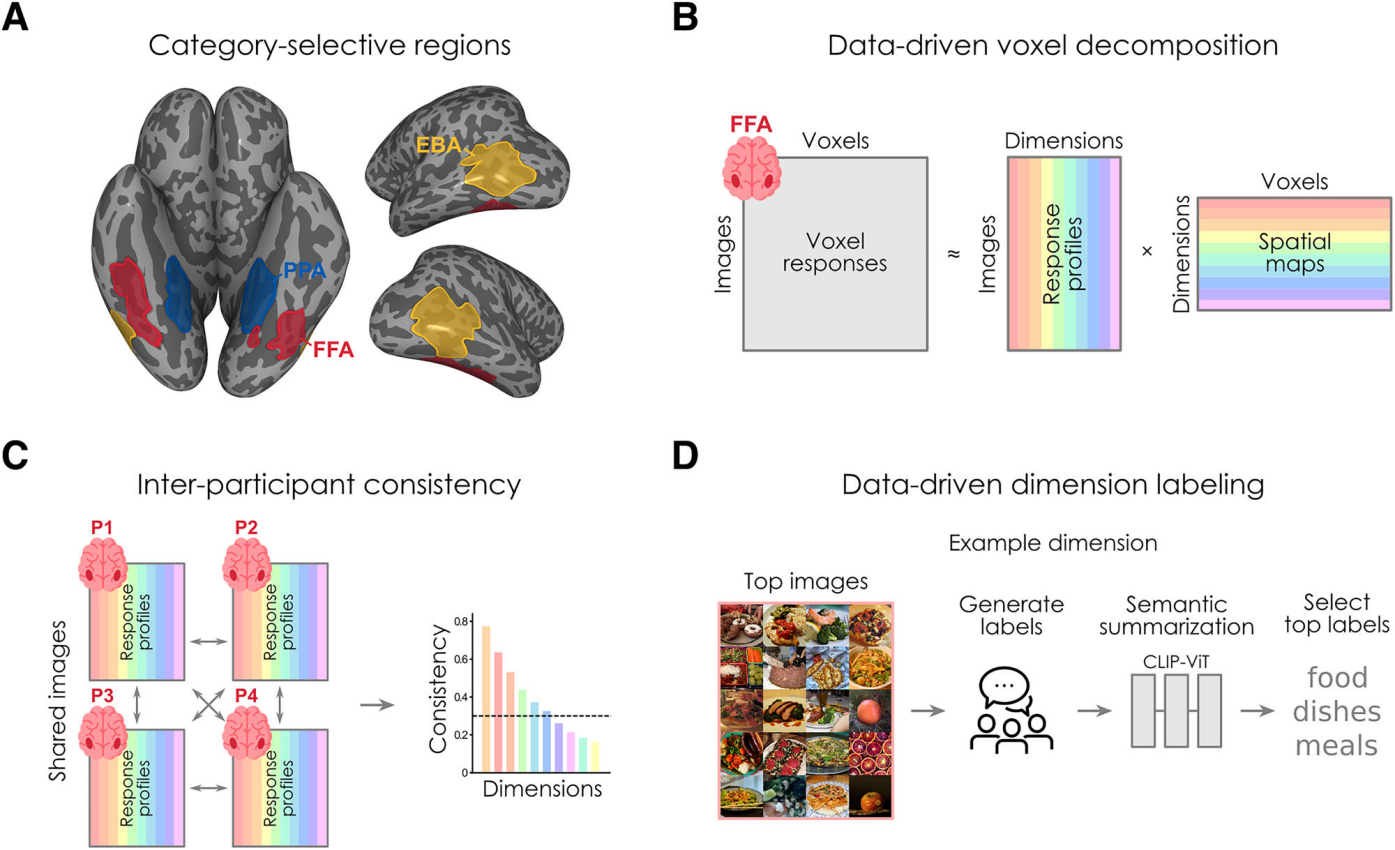
Data-driven fMRI voxel decomposition of category-selective areas. ***A***, Face-, body-, and scene-selective areas (FFA, EBA, and PPA) were defined with a functional localizer and are displayed on the inflated cortical surface for a representative participant (P1). ***B***, For each participant and area, voxel-wise responses were decomposed into latent dimensions with BNMF. Reliable dimensions were identified by repeating the decomposition, clustering solutions by response profile similarity, and taking cluster medians. ***C***, Across participants, dimensions were matched by correlating response profiles for shared images, and consistent dimensions were retained. ***D***, Dimensions were labeled via a behavioral task in which participants provided candidate labels for each dimension's highest-scoring images, followed by CLIP-ViT encoding of images and candidate labels to select the labels most aligned with each dimension.

A longstanding question is whether high-level visual cortex is best understood through category-selective areas or continuous feature maps. Here, we asked whether the categorical or dimensional view dominates or whether both reflect complementary expressions of a common underlying organization. To address this question, we used a data-driven decomposition of fMRI voxel responses from four participants viewing thousands of natural images from the NSD (Allen et al., 2022). We focused on three well-established category-selective areas: fusiform face area (FFA; Kanwisher et al., 1997), EBA (Downing et al., 2001), and parahippocampal place area (PPA; Epstein and Kanwisher, 1998; Fig. 1).

Within each area, we applied BNMF to decompose voxel-wise responses into latent dimensions (Schmidt et al., 2009; Khosla et al., 2022). Each dimension is defined by (1) a response profile, indicating how strongly it is activated by each image, and (2) a spatial map, indicating how strongly it contributes to each voxel. The decomposition explains each voxel’s response as a weighted sum of dimensions, aiming to separate mixed signals within a voxel. Since this method requires contributions to be non-negative, the dimensions combine additively, resulting in sparse and part-based representations that are easier to interpret.

This framework allowed us to distinguish among three possibilities. If the categorical view dominates, responses in each area should be captured by a small number of dimensions corresponding to the preferred category and clustering within that area. If the dimensional view dominates, responses should be captured by a common set of dimensions that span categorical boundaries and extend across cortex. If instead both views capture complementary aspects of a common underlying organization, responses should be captured by dimensions that encode the preferred category within a broader representational space and cluster within each category-selective area but extend as distributed maps across cortex.

To ensure robustness and generalizability, we used a multistep analysis pipeline. First, because BNMF can be performed at different granularities, we selected the optimal number of dimensions for each area using bi-cross-validation (Owen and Perry, 2009). Second, given that BNMF involves nonconvex optimization and can yield different solutions across runs, we repeated the decomposition multiple times, clustered dimensions based on their response profiles, and retained only dimensions that appeared reliably across runs. Third, since areas may encode different dimensions across individuals, we matched dimensions across participants by correlating their response profiles for a subset of shared images and selected those that were consistent. Finally, to interpret the remaining dimensions, we used a data-driven labeling procedure. In a separate behavioral experiment, participants (*N* = 38) viewed each dimension's highest-scoring images and freely generated candidate labels. To identify which candidate labels best summarized each dimension, we then encoded all images and candidate labels using CLIP-ViT, a deep learning model trained on hundreds of millions of image-text pairs (Radford et al., 2021). For each dimension, we computed the cosine similarity between each candidate label’s text embedding and all image embeddings, weighted by the response profile, and selected the highest-scoring labels.

Our analysis revealed that each category-selective area was characterized by a small set of dimensions that were highly consistent across individuals, alongside a larger set of less consistent dimensions. Despite strong univariate category selectivity (*t* > 2), each area was tuned to multiple dimensions. We identified eight consistent dimensions in FFA, 20 in EBA, and 10 in PPA (mean *r* across participant pairs ± SD; FFA: *r* = 0.46 ± 0.12; EBA: *r* = 0.49 ± 0.14; PPA: *r* = 0.45 ± 0.10), which together captured a substantial portion of voxel-response variance in each area (mean *R*^2^ across participants ± SD; FFA: *R*^2^ = 0.33 ± 0.06; EBA: *R*^2^ = 0.39 ± 0.01; PPA: *R*^2^ = 0.29 ± 0.09).

### Dimensions reveal multifaceted feature tuning in category-selective areas

**Figure 2.**
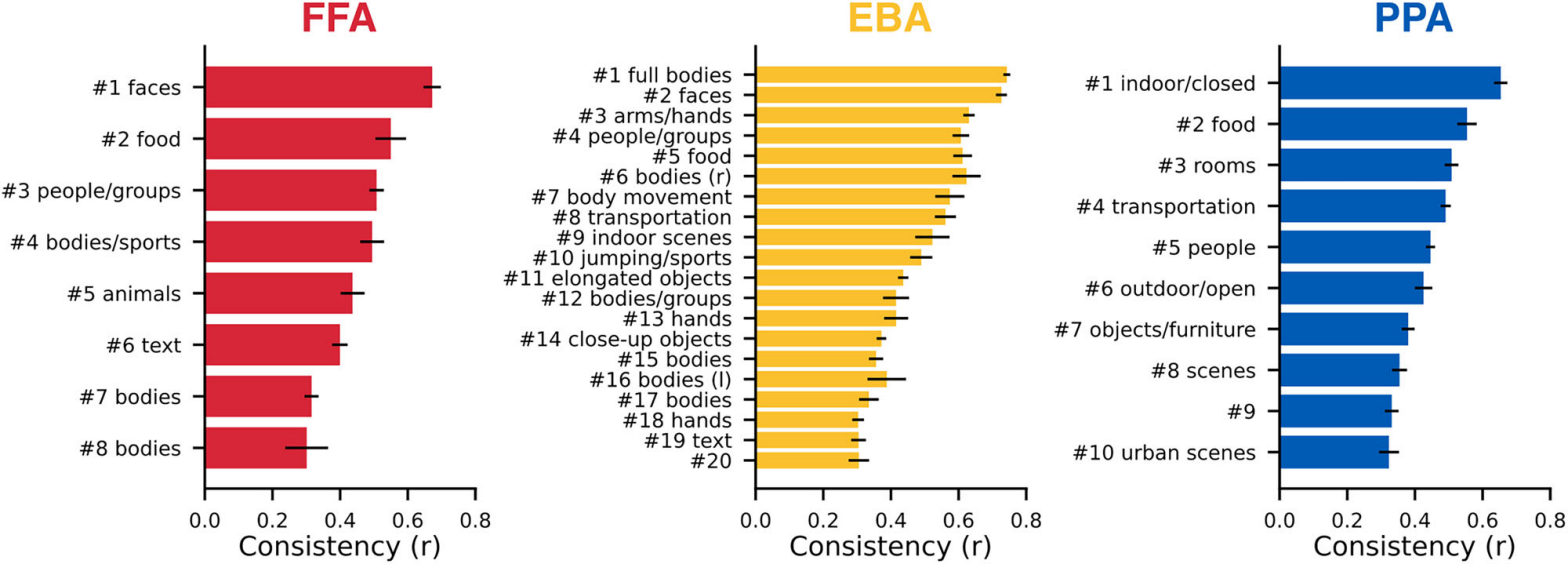
Mean pairwise correlation of consistent dimensions across participants in each category-selective area (±SEM). Dimensions were named based on the highest-scoring images and labels.

Having identified multiple consistent dimensions, we next examined their representational content and how they relate to known functional selectivities. The images mostly displayed shared semantic content, consistent with the established role of category-selective areas in view-invariant visual processing. The data-driven labeling procedure provided meaningful descriptive labels for most of the dimensions (FFA: 8/8; EBA: 19/20; PPA: 9/10; Fig. 2; Fig. S1), although a few remained ambiguous and were left unlabeled.

**Figure 3.**
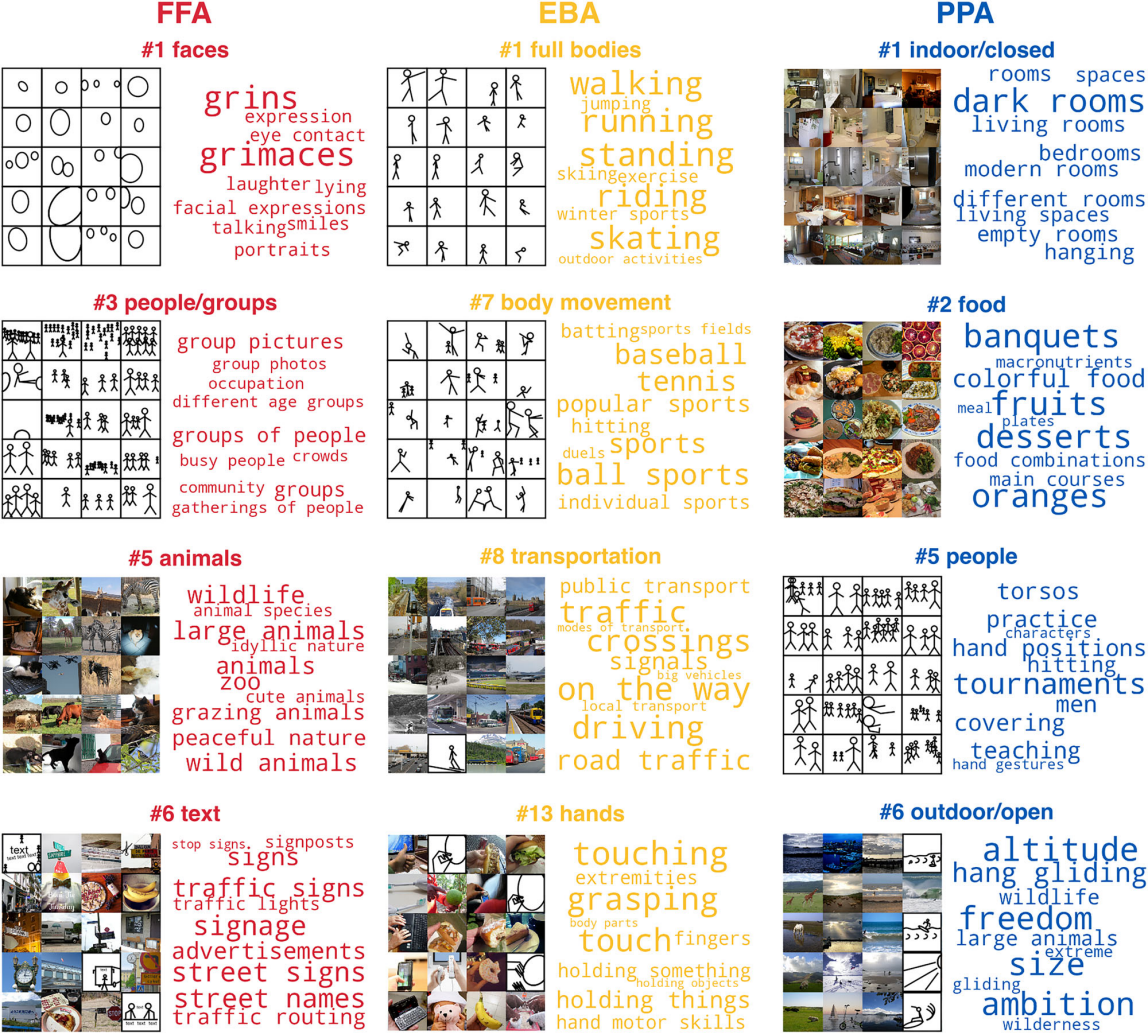
Interpretability of example dimensions from each category-selective area. Five highest-scoring images per fMRI participant and 10 highest-scoring labels based on the behavioral participants. Images of people were replaced with stick figures.

As expected, the most consistent dimensions in each area were related to its preferred category: faces in FFA, whole bodies in EBA, and scenes in PPA (Fig. 3). However, the dimensions were not limited to these broad categories and also captured finer within-category features. In EBA, for example, dimensions differentiated body parts, including full bodies, arms, hands, and faces, consistent with its role in encoding fine-grained body information (Bracci et al., 2010, 2015; Orlov et al., 2010; Downing and Peelen, 2011; Weiner and Grill-Spector, 2011; Ramirez et al., 2024). Similarly, in PPA, dimensions encoded scene properties such as openness and naturalness, distinguishing indoor from outdoor, enclosed from open, and urban from natural environments (Kravitz et al., 2011; Park et al., 2011; Lescroart and Gallant, 2019). In contrast, in FFA, dimensions did not capture within-category distinctions among specific facial parts, as reported in more controlled studies (Henriksson et al., 2015; de Haas et al., 2021), which may be due to the limited variability and smaller size of faces in natural images. However, FFA also contained an animal dimension, which may reflect sensitivity to animal faces or broader animacy-related features (Kanwisher et al., 1999; Sha et al., 2015).

Beyond dimensions corresponding to each area's preferred category, additional dimensions captured meaningful content from other domains. In FFA, while some dimensions reflected additional people-related concepts, such as groups, bodies, and sports, others extended to animals, food, and text. In EBA, dimensions captured not only additional body-related concepts, such as movement and sports, but also food, transportation, indoor scenes, close-up objects, and text. In PPA, alongside additional scene-related concepts, such as transportation and objects/furniture, dimensions also represented food and people. These nonpreferred dimensions were less consistent across participants, which might explain why earlier studies were less likely to detect them. Moreover, the diversity of dimensions differed substantially across areas, with EBA and PPA showing more heterogeneous tuning than FFA. Most of the identified dimensions reflected high-level semantic content and showed little sensitivity to low- or mid-level visual features such as shape, texture, or color. A partial exception was observed in EBA, where certain body-related dimensions were sensitive to the spatial location of bodies on the left or right side of the image, reflecting potential influences of retinotopic organization.

A potential concern is that the dimensions found in each area may reflect a single, category-selective continuum rather than distinct features. For example, in FFA, multiple dimensions could primarily reflect a single “faceness” continuum (e.g., faces > bodies > text), instead of distinct feature axes (e.g., for faces, bodies, and text). To test this possibility, we used cosine similarity to quantify how similar the response profiles of each dimension were within an area. We compared dimensions related to the area's preferred category (e.g., face-related dimensions in FFA, such as faces, bodies, and animals) with dimensions related to nonpreferred categories (e.g., nonface dimensions in FFA, such as food and text). If the dimensions mainly sample a single continuum, then all dimensions should align with the same dominant axis, producing uniformly high similarity. In contrast, if an area encodes additional, partially independent feature axes, similarity should be reduced.

Using the raw response profiles, similarities were uniformly high both within and between groups (all mean cosine similarities >0.64). This suggests that many dimensions share a response component across stimuli. However, this global component could simply reflect an overall preference for certain images (e.g., high-contrast or face-like images) that would inflate similarity even if the underlying organization is not well described by a single continuum. To focus on similarity beyond this global component, we subtracted the mean response profile across images from each dimension and recomputed similarities. After mean-centering, similarities dropped substantially. Between-group similarity was low (FFA: *M* = −0.13 ± 0.02; EBA: *M* = −0.11 ± 0.01; PPA: *M* = −0.14 ± 0.01) and even within-group similarity was low for both preferred dimensions (FFA: *M* = −0.14 ± 0.05; EBA: *M* = 0.01 ± 0.01; PPA: *M* = −0.02 ± 0.03) and for nonpreferred dimensions (FFA: *M* = −0.13 ± 0.01; EBA: *M* = 0.02 ± 0.01; PPA: *M* = −0.10 ± 0.01). These results argue against a single category-selective continuum and instead suggest complementary dimensions, consistent with multidimensional tuning.

Together, these findings suggest that although category-selective areas showed strong selectivity for their preferred category in univariate analyses, they exhibited both finer within-category and coarser between-category distinctions in multivariate analyses, underscoring their rich functional organization.

### Multidimensional tuning persists in highly category-selective voxels

So far, our results indicate that category-selective areas are tuned to multiple dimensions beyond their preferred category. However, since these areas were defined with coarse functional localizer contrasts (e.g., faces > all other categories), they likely included voxels with only weak category preference under broader stimulus sampling. This raised the possibility that the observed multidimensional tuning could be at least partly driven by the inclusion of weakly selective voxels, rather than the intrinsic tuning of category-selective cortex.

This alternative explanation provided a critical test: if voxels with strong category selectivity exhibit narrow tuning to one (or a few) category-aligned dimensions, with little or even negative tuning to all other dimensions, then multidimensionality should be mainly due to the inclusion of less selective voxels. Conversely, if stronger category selectivity is associated with tuning to multiple dimensions, this would support a genuinely multidimensional code even in the most category-selective voxels. To evaluate this possibility, we computed a category selectivity index (*d′*) for each voxel using the preferred localizer contrast (e.g., faces > all other categories in FFA), quantifying how strongly each voxel responded to its area's preferred category relative to all others. We also computed a sparseness index for each voxel (Hoyer, 2004), where higher values indicate that responses are concentrated on few dimensions and lower values indicate that responses are distributed across multiple dimensions.

**Figure 4.**
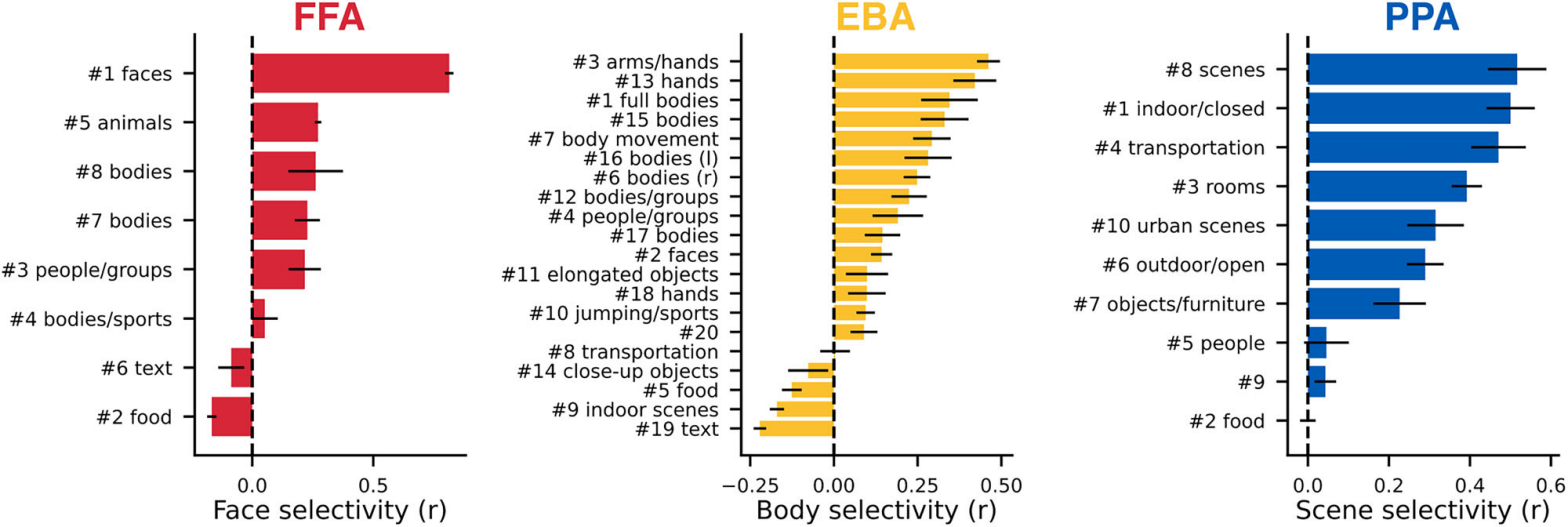
Mean correlation of voxel-wise category selectivity based on the functional localizer and dimension tuning based on NSD (±SEM).

Across all areas, category selectivity showed little to no association with sparseness (mean *r* across participants ± SD; FFA: *r* = 0.23 ± 0.15, *p* = 0.06; EBA: *r* = −0.03 ± 0.07, *p* = 0.46; PPA: *r* = −0.01 ± 0.08, *p* = 0.77), indicating that more selective voxels were not tuned to substantially fewer dimensions. Moreover, the relationship between category selectivity and tuning to individual dimensions confirmed that even highly category-selective voxels responded to multiple dimensions, primarily capturing the preferred category but also complementary features (Fig. 4). For example, highly face-selective voxels in FFA also responded to dimensions related to bodies and animals. Highly body-selective voxels in EBA were additionally tuned to actions, faces, and elongated objects. Highly scene-selective voxels in PPA also responded to transportation, objects/furniture, and people. These findings further support more flexible notions of category selectivity, which allow for tuning to multiple features related to the preferred category (Op de Beeck et al., 2008; Mahon and Caramazza, 2009, 2011; Grill-Spector and Weiner, 2014).

Together, these results show that multidimensional tuning cannot be explained merely by weak category selectivity. Instead, even the most category-selective voxels encode multiple dimensions, consistent with a multifaceted code.

### Dimensions from category-selective areas explain activity throughout high-level visual cortex

Having characterized the representational content of the dimensions, we next asked whether they are spatially clustered within each area or spatially distributed across cortex. To test this, we evaluated how well dimensions derived from each area predicted voxel responses to novel images across the entire cortex.

**Figure 5.**
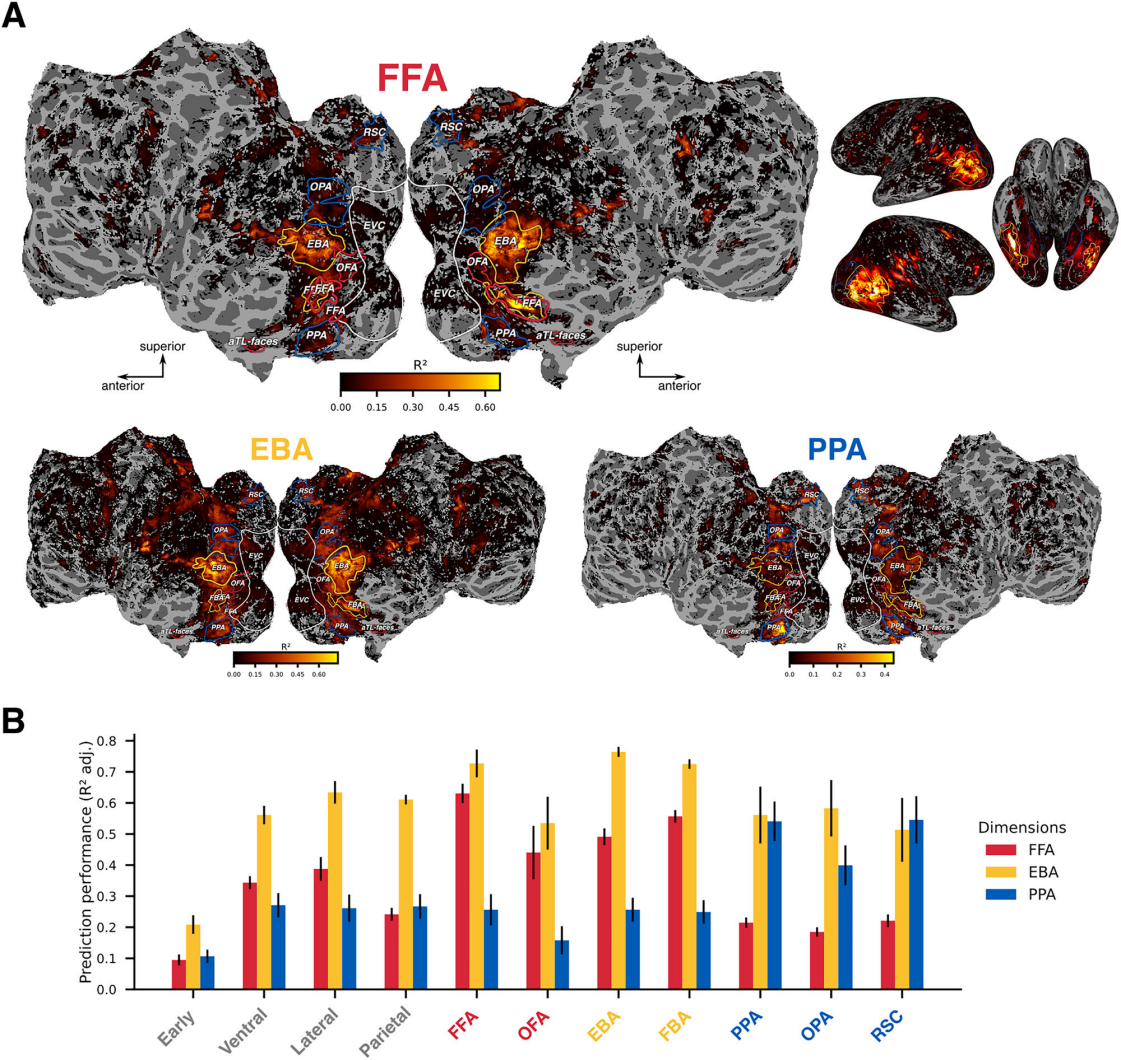
Prediction performance of dimensions from category-selective areas across cortex. ***A***, Voxel-wise prediction performance (*R*^2^) for dimensions from each area projected onto the flattened cortical surface for a representative participant (P1). Voxels thresholded at *p* < 0.01 (one-sided, FDR-corrected) based on permutation tests. ***B***, Mean prediction performance (noise ceiling-adjusted *R*^2^) for different areas, averaged across participants (±SEM). Note that absolute differences partly reflect the number and diversity of dimensions, whereas relative differences reveal the expected functional preferences.

First, we used responses from each category-selective area to an independent set of test images and expressed them as combinations of that area's learned spatial maps, using non-negative least squares regression. This produced dimension response profiles for these new test images. Next, we built voxel-wise encoding models across cortex using ridge regression, with the response profiles as predictors and the corresponding voxel-wise responses across the entire cortex as targets. Finally, for each area's set of dimensions, we computed and mapped cross-validated variance explained (*R*^2^) across cortex (Fig. 5, Fig. S2). If a given set of dimensions predicts responses only within the category-selective area from which it was derived, this would support a localized organization. Conversely, if dimensions derived from one area predict responses in other areas, this would support a distributed organization.

For each set of dimensions, prediction performance peaked in high-level visual cortex but also extended partly into early visual and prefrontal cortex. Within each category-selective area, locally derived dimensions explained responses especially well, often approaching the estimated noise ceiling. Crucially, however, the dimensions also explained responses across the broader high-level visual cortex. For example, FFA-derived dimensions predicted responses in body-selective areas such as EBA and FBA, whereas EBA-derived dimensions predicted responses in face-selective areas such as FFA and superior temporal sulcus (STS). Similarly, EBA-derived dimensions accounted for responses in scene-selective areas, including PPA and occipital place area (OPA). Since EBA-derived dimensions were more numerous and diverse than those derived in FFA or PPA, these dimensions explained the largest absolute amount of variance. However, when examining relative prediction performance, each set of dimensions showed the strongest predictivity in areas that matched its functional preferences. While some cross-region prediction may reflect spatial proximity between neighboring areas (e.g., FFA and FBA, EBA and OPA), the magnitude and selectivity of these effects suggest that shared dimensions, rather than categorical boundaries alone, underlie the organization of category-selective cortex.

Together, these findings indicate that the dimensions capture responses within category-selective areas while also predicting responses across high-level visual cortex. This suggests that category-selective areas are embedded in broader functional networks that encode both distinct and shared features.

### Dimensions form distinct clusters within category-selective areas but extend as distributed maps across cortex

**Figure 6.**
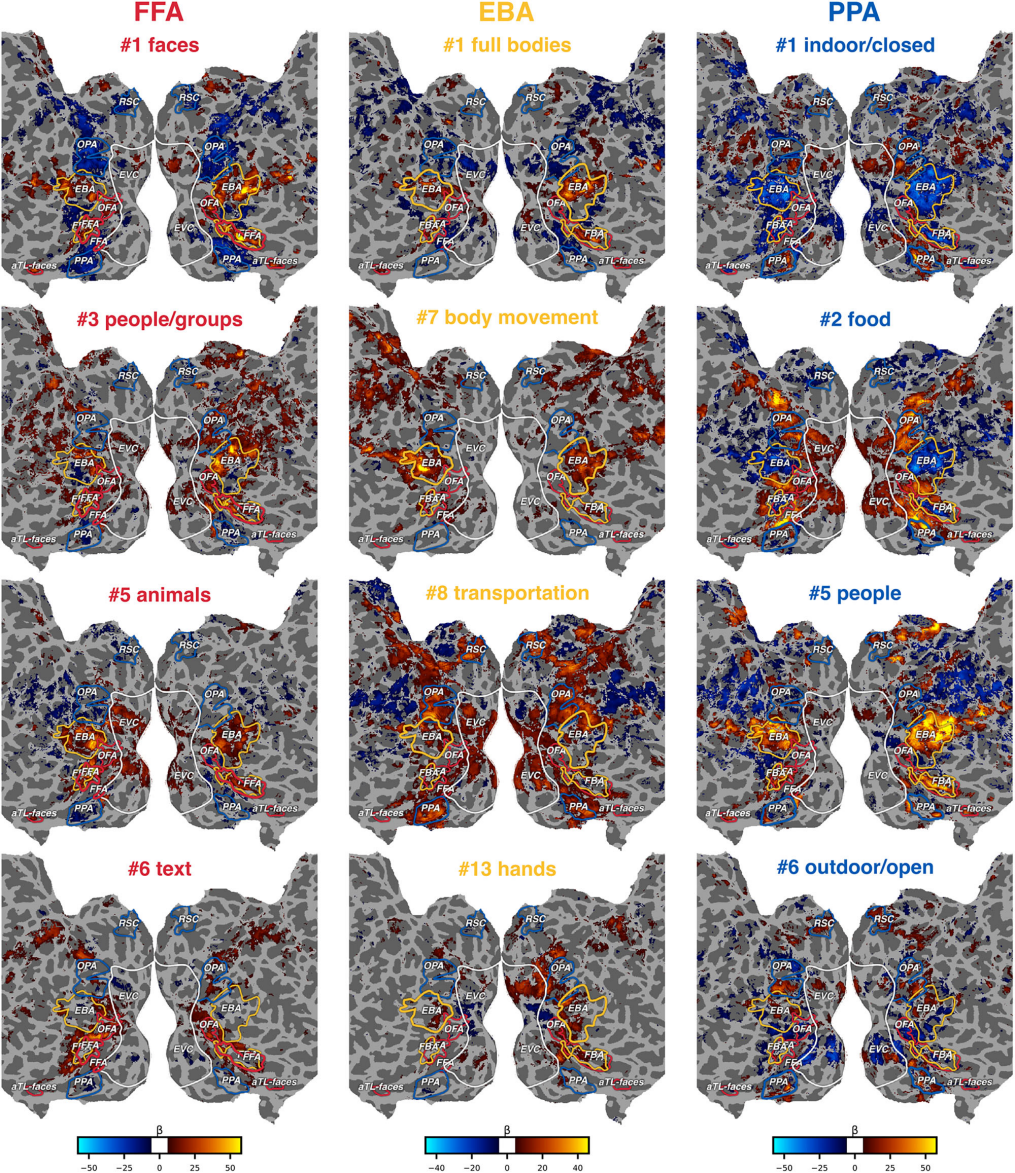
Voxel-wise tuning maps for example dimensions from each category-selective area projected onto the flattened cortical surface for a representative participant (P1). Voxels thresholded at *p* < 0.01 (one-sided, FDR-corrected) based on permutation tests.

Having established that dimensions derived from category-selective areas predict responses across high-level visual cortex, we next asked how individual dimensions are spatially organized. If dimensions within an area overlap strongly, this would suggest that the same neural population encodes multiple dimensions. If instead dimensions form distinct spatial clusters, this would suggest that distinct neural populations encode different dimensions. To distinguish between these possibilities, we examined the spatial maps for each dimension from the encoding models (Fig. 6, Figs. S3–S6).

Many dimensions showed the expected selectivity patterns: face-related dimensions were concentrated in face-selective areas, body-related dimensions in body-selective areas, and scene-related dimensions in scene-selective areas. Importantly, most dimensions formed distinct clusters within each category-selective area, revealing a finer-grained functional organization. In FFA, clusters were tuned to nonface features (Çukur et al., 2013). In EBA, clusters encoded different body parts, including full bodies, faces, arms, and hands (Bracci et al., 2010, 2015; Orlov et al., 2010; Downing and Peelen, 2011; Ramirez et al., 2024). In PPA, clusters encoded different scene properties, such as openness and possibly distance (Lescroart and Gallant, 2019). These findings support an account in which category-selective areas comprise multiple tuning maps (Op de Beeck et al., 2008; Mahon and Caramazza, 2009, 2011; Grill-Spector and Weiner, 2014). This also extends earlier reports that category-selective areas encode nonpreferred features (Haxby et al., 2001; Cichy et al., 2012), demonstrating that such information is not only present but also spatially organized.

Beyond local clusters, many dimensions formed distributed maps across high-level visual cortex and reappeared in several category-selective areas, consistent with large-scale, partially overlapping feature maps. For example, an EBA-derived face dimension reappeared in face-selective areas, including FFA, STS, OFA, an anterior temporal face area (aTL-faces), and a prefrontal face area near inferior frontal sulcus (Tsao et al., 2008; Nikel et al., 2022). Similarly, people-related dimensions were mostly shared between EBA and FFA, supporting the existence of interconnected face- and body-selective networks (Taubert et al., 2022; van Dyck and Dobs, 2026). In addition, body-related dimensions appeared in and around multiple scene-selective areas, consistent with recently described body-selective areas (Zhao et al., 2025). This distributed layout also accounts for the commonly observed spatial overlap between neighboring category-selective areas. For example, in FFA, a text-related dimension extended into visual word form area (VWFA), and body-related dimensions overlapped with FBA. Other dimensions, such as those tuned to food, were even more broadly distributed, spanning multiple areas like V4, FFA, and PPA (Henderson et al., 2025). Consistent with established lateralization patterns, face- and body-related dimensions were predominantly right-lateralized, whereas text-related dimensions were mostly left lateralized (Willems et al., 2010; Dehaene and Cohen, 2011; Behrmann and Plaut, 2015). Overall, these cortical topographies suggest an organization that combines locally selective clusters with globally distributed maps across high-level visual cortex (Weiner and Grill-Spector, 2010; Behrmann and Plaut, 2013; Miyakawa et al., 2018; Contier et al., 2024; Bogdan et al., 2025; Ritchie et al., 2026).

In summary, these results refine our understanding of the functional organization in high-level visual cortex. Individual dimensions form distinct clusters within category-selective areas yet also participate in continuous feature maps across cortex. This combination of local selectivity and global distribution provides a unified framework for how high-level visual cortex balances specificity and flexibility.

## Discussion

We asked whether high-level visual cortex is organized around category-selective areas or continuous feature maps and whether these reflect competing views or complementary expressions of a common underlying organization. Our data-driven analysis of fMRI responses suggests that high-level visual cortex is organized along multiple representational dimensions, with elements of both categorical and dimensional accounts. Despite being derived directly from cortical responses to complex natural images, the resulting dimensions were interpretable and revealed both known selectivities and potentially novel ones. Category-selective areas were well characterized by dimensions that included both category-related features and more diverse features. Many dimensions aligned with each area's category preference, consistent with domain-specific coding (Downing et al., 2005; Kanwisher, 2010). At the same time, the areas also reflected finer within-category and coarser between-category distinctions, indicating multifaceted representations. This pattern is in line with recent evidence that category-selective cortex flexibly encodes both domain-specific and domain-general information (Khosla and Wehbe, 2023; Vinken et al., 2023).

Combining this decomposition with encoding models linked functional organization across spatial scales, from feature-selective clusters to category-selective areas to continuous feature maps. Within category-selective areas, dimensions formed distinct clusters, consistent with a mosaic of feature-selective neural populations. Beyond these areas, many dimensions extended as distributed maps across high-level visual cortex, consistent with broader functional networks. Because the learned dimensions were continuous yet constrained to be non-negative, they allowed us to capture both sharply selective peaks within areas and smooth gradients across cortex. Together, these observations point to a hierarchical organization in which fine-scale subclusters are tuned to specific dimensions, category-selective areas sample subsets of these dimensions, and large-scale feature maps emerge from broadly overlapping dimensions.

Our findings resonate with a growing body of work that uses encoding models to reveal fine-grained functional organization in the brain (Çelik et al., 2021; Efird et al., 2024; Luo et al., 2024; Doerig et al., 2025; Hwang et al., 2025; Wasserman et al., 2025). The topographies of individual dimensions closely resembled those of behavior-derived object dimensions (Hebart et al., 2020; Contier et al., 2024), suggesting that these neural representations may support goal-directed behavior. Importantly, previous work has shown that category selectivity arises from sparse tuning to a limited set of high-level dimensions (Contier et al., 2024; Ritchie et al., 2026). This is also consistent with evidence linking category selectivity to sparsely distributed neural codes and connectivity-based constraints (Weiner and Grill-Spector, 2010; Behrmann and Plaut, 2013; Osher et al., 2016; Miyakawa et al., 2018; Op de Beeck et al., 2019; Molloy et al., 2024).

Notably, alternative accounts emphasize dense high-dimensional feature spaces of low- or mid-level visual properties, with category selectivity emerging as a byproduct of broadly distributed coding (Andrews et al., 2015; Henderson et al., 2023; Vinken et al., 2023). Our findings do not rule out the possibility that the dimensions identified here themselves arise from such representations at finer spatial scales. At the resolution of fMRI, however, the response profiles of category-selective areas are well captured by a small number of higher-level, interpretable dimensions that differ in their representational content and cortical topography. This multidimensional tuning is consistent with recent proposals of region-based feature coding, in which neurons encode receptive fields in a semantic feature space and thereby link categorical and feature-based coding (Cao et al., 2025).

Recent computational work offers a mechanistic explanation for how such organization might arise. Contrastive learning models, for instance, can produce brain-like functional organization without explicit category-specific mechanisms (Prince et al., 2024), whereas topographic neural network models provide a framework for understanding how overlapping feature tuning generates spatial patterns that integrate selectivity across scales (Blauch et al., 2022, 2025; Doshi and Konkle, 2023; Margalit et al., 2024; Lu et al., 2025).

Despite its contributions, our approach has several limitations. First, the decomposition imposed a non-negativity constraint, which is well suited to capturing excitatory BOLD responses but is insensitive to potentially informative inhibitory signals (Pérez-Ortega et al., 2024). Although we relaxed this constraint in the subsequent encoding models, future work could develop more flexible methods that explicitly capture both excitatory and inhibitory components. Second, the non-negativity constraint imposed by NMF encourages sparser solutions overall, which may limit the interpretability of absolute sparseness values. We therefore restricted the use of the sparseness index to quantifying relative differences in tuning concentration across voxels within a given area and do not interpret absolute sparseness levels as direct evidence for sparse coding at the regional level. Third, not all dimensions were easily interpretable, reflecting many-to-one mappings between visual features and neural responses. Accordingly, the descriptive labels from the data-driven labeling procedure should be viewed as hypotheses rather than definitive interpretations. Finally, as with any data-driven approach, the recovered dimensions are shaped by the stimulus set. Limited diversity or systematic biases in the natural images may shape which dimensions emerge (Shirakawa et al., 2025), although our emphasis on robustness and generalizability likely reduced the impact of spurious effects.

Our findings point to several promising directions for future research. A central question is whether these dimensions actively guide behavior and how flexibly they adapt to changing task demands (Peelen and Downing, 2017; Bracci and Op de Beeck, 2023; Ritchie et al., 2026). Systematically testing their stability across tasks, datasets, and imaging modalities will clarify how general or task dependent these representations are. Future work should also investigate how these dimensions interact across areas (Op de Beeck et al., 2008) and evolve over time (Teichmann et al., 2024; Shi et al., 2026), potentially revealing their computational roles within larger networks. Another priority is to probe the granularity of these dimensions to better understand how fine- and coarse-scale representations coexist (Gauthaman et al., 2025; Han and Bonner, 2025). While our decomposition revealed mixed selectivity at the voxel level, more discrete tuning may exist at finer spatial scales (Quiroga et al., 2005), highlighting the need for higher-resolution methods to uncover potentially hidden structure. Finally, comparing biologically derived dimensions to those learned by artificial neural networks may uncover shared representational strategies between biological and artificial visual systems (Kanwisher et al., 2023; Hosseini et al., 2024; Chen and Bonner, 2025; Mahner et al., 2025).

Our results reveal that category-selective areas and continuous feature maps may reflect the same underlying functional organization. Within each area, voxels are characterized by interpretable dimensions that capture both fine within-category distinctions and broader between-category distinctions. These dimensions cluster within category-selective areas but also extend as distributed maps across cortex. Together, these findings show that categorical and dimensional views need not be viewed as competing accounts but rather as complementary perspectives on the same underlying organization. This may help the visual system balance representational specificity and flexibility across a wide range of perceptual and cognitive functions.

## Data Availability

The data supporting our analyses were obtained from the publicly available Natural Scenes Dataset (http://naturalscenesdataset.org/). The Python code (version 3.8.20) used for data analysis and visualization is publicly available on GitHub (https://github.com/levandyck/roidims).
